# Leadership, safety culture and patient safety in hospitals: in search of evidence

**DOI:** 10.1186/1472-6963-14-S2-P64

**Published:** 2014-07-07

**Authors:** Oliver Kessler

**Affiliations:** 1 School of Business, Lucerne University of Applied Sciences and Arts, Lucerne, Switzerland

## Background

At least since the publication of “To Err is Human” in the year 2000 we all know that hospitals could be safer than they are [1]. In the meantime the knowledge about patient safety and evidence based safety practices grew substantially but too often these practices do not reach the patients [2]. Evidence based medicine, nursing and therapy are advancing but the implementation gap seems also to be growing [3]. Are evidence based leadership and an appropriate safety culture the solution to this implementation gap since “more than enough evidence exists to prompt decisive action” [4]? Do we suffer blind spots on the roles of leadership and safety culture? The first objective of this study was to review theories, models and empirical evidence of the functions, roles and interdependences of leadership practices, safety cultures and patient safety outcomes in hospitals. Secondly, empirical studies will be conducted to test and validate the framework.

## Materials and methods

Various databases and gray literature have been searched and the selected publications systematically reviewed. A framework for evidence based leadership has been developed as well as discussed with and validated by patient safety experts and organizational scientists.

## Results

The theoretical model derived from the literature and the workshops shows the respective influences and interdependences between leadership practices, safety cultures and patient safety outcomes. A framework for evidence based leadership has been developed.

## Conclusions

The model seems to be functional as a framework for empirical studies to analyse the influences and interdependences between local leadership practices, safety cultures and patient safety outcomes.
